# Clinicopathological Characteristics and Outcomes of Patients With Endometriosis-Related Hemorrhagic Ascites: An Updated Systematic Review of the Literature

**DOI:** 10.7759/cureus.26222

**Published:** 2022-06-22

**Authors:** Anastasios Pandraklakis, Anastasia Prodromidou, Dimitrios Haidopoulos, Anna Paspala, Maria D Oikonomou, Nikolaos Machairiotis, Alexandros Rodolakis, Nikolaos Thomakos

**Affiliations:** 1 First Department of Obstetrics and Gynecology, Alexandra Hospital, National and Kapodistrian University of Athens, Athens, GRC; 2 Third Department of Surgery, Attikon University Hospital, National and Kapodistrian University of Athens, Athens, GRC; 3 Homerton Fertility Centre, Homerton University Hospital NHS Trust, London, GBR; 4 Department of Obstetrics and Gynaecology, Accredited Endometriosis Centre, Northwick Park Hospital, London North West University Healthcare, London, GBR

**Keywords:** hemorrhagic, ascites, hemoperitoneum, ovarian cancer, endometriosis

## Abstract

The presence of ascites is a common clinical presentation in gynecologic oncology patients. Hemorrhagic ascites (HA) due to endometriosis is a rare presentation that can be easily misdiagnosed as ovarian malignancies. The present study aims to update the currently available knowledge on the characteristics of patients presenting with HA due to endometriosis.

A systematic search was conducted for articles published from January 2000 to July 2020 using the Medline, Scopus, and Google Scholar databases along with the references of the full-text articles retrieved. Papers describing cases of women over 18 years with or without previous history of endometriosis were assessed. Only cases with histologically proven hemorrhagic ascites of endometriosis origin were included.

Twenty-nine studies (27 case reports and two case series) comprising 32 patients were evaluated. The mean patients’ age was 32 years, while six of the patients had a previous history of endometriosis. The mean amount of drained ascitic fluid was 4,200 mL, whereas three patients underwent thoracentesis due to pleural effusions. The treatment options included not only medical but also surgical therapies. Fertility preservation was achieved in 27 patients, while two of them achieved pregnancy with in vitro fertilization (IVF) techniques.

Endometriosis-related hemorrhagic ascites is a relatively rare expression of the disease. Endometriosis-related hemorrhagic ascites should be considered in the differential diagnosis (DD) of women with ascites and clinical suspicion of endometriosis. The available literature is limited to case reports and case series and thus indicates further research in the field to decode the pathophysiology of the disease and decide on the optimal treatment.

## Introduction and background

Ascites is the accumulation of fluid in the peritoneal cavity and are typically presented with abdominal distension, tenderness, dyspnea, and fatigue [1]. The differential diagnosis (DD) of ascites is complicated by atypical symptoms and the wide variety of diseases included and thus disabling the final diagnosis [2]. In that setting, the most common cause of ascites is hepatic cirrhosis due to portal hypertension, which accounts for approximately 80% of ascites DD [3]. Among the other causes, peritoneal disease (cancerous, infectious, or inflammatory), hypoalbuminemia (nephrotic syndrome), and rare conditions (chylous, pancreatic, urinary, and hemoperitoneum) have also been reported in the etiology of peritoneal fluid concentration [4]. Hemorrhagic (or bloody) ascites have been reported as the presence of red blood cells (RBC) ≥ 10,000 per mm^3^, while in dark blood-colored ascitic fluid, about 50,000 RBCs per mm^3^ have been measured [5].

From the point of view of gynecology, ascites is a frequent presentation in women with ovarian malignancies investigated in gynecologic oncology clinics [6]. In addition to this, there are also various benign gynecologic diseases that have been characterized by the presence of ascites, including ovarian hyperstimulation syndrome, Meigs syndrome, benign ovarian tumors, fibroids, and endometriosis, which makes the final diagnosis difficult to be established [7]. Paracentesis and cytological examination of the ascitic fluid is a simple procedure but with limited diagnostic accuracy.

Endometriosis is a common benign gynecologic disorder that is mainly found in women of reproductive age and is defined as the presence of endometriotic tissue in areas outside the uterine cavity [8]. The pelvic structures and organs are the most prevalent sites of endometriosis despite the fact that in rare cases endometriotic lesions can grow in extrapelvic sites [9]. Hemorrhagic ascites (HA) associated with endometriosis is a rare entity that creates diagnostic dilemmas for gynecologists and complicates the management of the disease.

The aim of the present study was to update the currently available knowledge on the characteristics of patients presenting with HA due to endometriosis. More specifically, given the lack of specific guidelines and consensus on the appropriate management, we sought to investigate the potential mechanisms of endometriosis-related ascites formation, clinical presentation, and disease characteristics, as well as the type of interventions for the management of the disease and postoperative outcomes.

## Review

Materials and methods

Study Design and Eligible Studies

The present systematic review was performed in accordance with the guidelines of the Preferred Reporting Items for Systematic Reviews and Meta-Analyses (PRISMA) according to the authors’ predetermined inclusion criteria [10]. Three authors (APr, APan, and NT) independently and meticulously searched the literature, excluded overlaps, and structured the tables with the selected indices. All appropriate observational studies (prospective and retrospective) and case reports and case series of patients with a diagnosis of endometriosis-related HA were considered eligible for inclusion in the present study. The cases with hemorrhagic peritoneal fluid due to endometriosis were considered eligible, while cases of hemoperitoneum related to rupture of ovarian endometrioma or other endometriotic nodules were excluded. Additionally, those reported respective cases of HA and hemoperitoneum during pregnancy were also not included. Cases describing the identification of ascetic fluid in which the paracentesis revealed “yellow” fluid were also not included. Review articles, conference papers, abstracts, letters to the editor, and animal studies were excluded from analysis and tabulation. Additionally, video articles that were accompanied by abstracts with insufficient data were also excluded. Only articles written in the English language were included.

Search Strategy and Data Collection

**Figure 1:**
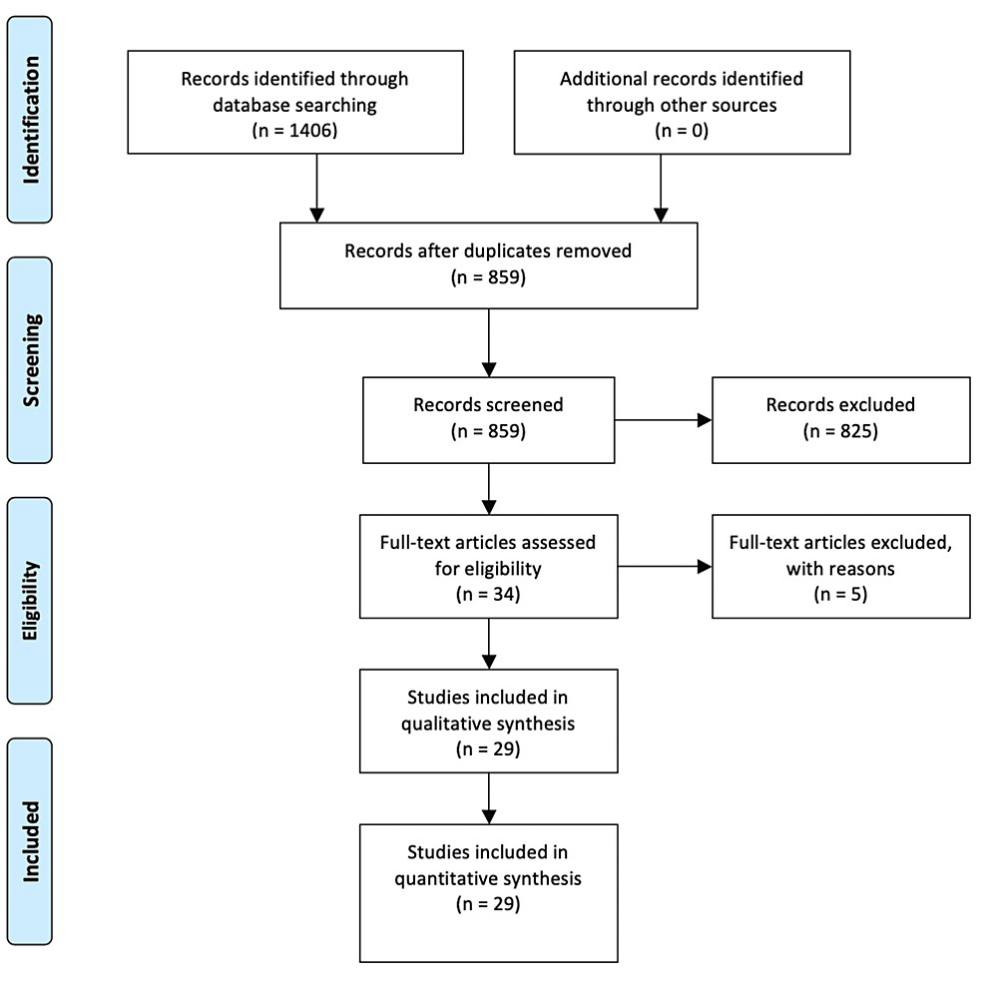
Search flow diagram

We performed a meticulous and systematic search of the literature for articles published from January 2000 to July 2020 using the Medline (2000-2020), Scopus (2000-2020), and Google Scholar (2000-2020) databases along with the references of the articles that were retrieved in full text. The following keywords were used for the search: “endometriosis,” “hemorrhagic ascites,” “hemoperitoneum,” and “bloody ascites.” A minimum number of search keywords were utilized in an attempt to assess an eligible number that could be easily searched while simultaneously minimizing the potential loss of articles. Articles that fulfilled or were deemed to fulfill the inclusion criteria were retrieved; all articles describing cases of women aged >18 years with or without previous history of endometriosis who were diagnosed with HA that was histologically proven to be of endometriosis origin were included. The PRISMA flow diagram schematically presents the process of article selection (Figure 1).

Our search strategy included the following MeSH terms: (“blood” (MeSH Subheading) OR “blood” (All Fields) OR “blood” (MeSH Terms) OR “bloods” (All Fields) OR “haematology” (All Fields) OR “hematology” (MeSH Terms) OR “hematology” (All Fields) OR “haematoma” (All Fields) OR “hematoma” (MeSH Terms) OR “hematoma” (All Fields) OR “haemorrhage” (All Fields) OR “hemorrhage” (MeSH Terms) OR “hemorrhage” (All Fields) OR “haemorrhages” (All Fields) OR “hemorrhages” (All Fields) OR “haemorrhagic” (All Fields) OR “haemorrhaging” (All Fields) OR “hematologies” (All Fields) OR “haematomas” (All Fields) OR “hematomas” (All Fields) OR “hematomas” (All Fields) OR “hematomae” (All Fields) OR “hemorrhaged” (All Fields) OR “hemorrhagic” (All Fields) OR “hemorrhagical” (All Fields) OR “hemorrhaging” (All Fields)) AND (“ascite” (All Fields) OR “ascites” (MeSH Terms) OR “ascites” (All Fields) OR “ascitic” (All Fields)) AND (“endometriosis” (MeSH Terms) OR “endometriosis” (All Fields) OR “endometrioses” (All Fields)).

Outcomes Retrieved

The management of the disease and recurrences and reoperation rates during follow-up were set as the main outcomes of the present study. Concerning the secondary findings of our study, the characteristics of the disease, including the concomitant presence of pleural effusion, clinical presentation and symptomatology, type of diagnostic procedure, amount of fluid drained recurrence rates, and follow-up after the last treatment, were appraised. Additionally, levels of CA 125 (for studies with multiple values, we considered the highest one) and hemoglobin were evaluated. Data on patient characteristics included age, ethnicity, parity, and gravidity of women.

Definitions

Hemorrhagic ascites is defined as the detection of more than 10,000 red blood cells (RBC) per μL in the ascitic fluid. However, when RBC count in the ascitic fluid was not available, the diagnosis of HA was based on the radiographic findings and/or macroscopic appearance of the bloody/dark red color of the fluid drained.

Quality Assessment

Case reports and case series are associated with elevated bias due to the nature of those types of studies [11]. Nonetheless, in the case where data on a certain condition is limited, evidence from those studies is considered of clinical importance. We evaluated the quality of the enrolled studies by adopting the quality assessment tool for case reports and case series proposed by Murad et al. [11]. More specifically, the methodological quality of the studies was assessed based on the criteria, including the domains of ascertainment, causality, selection, and reporting. The sum of the scores derived from eight critical questions that referred to the domains was used to evaluate the quality of each study and the reviewer’s judgment on the presence of the most important domains according to a certain clinical case.

Statistical Analysis

Continuous variables were interpreted as median and range, while categorical variables as frequencies and percentages. The level of statistical significance was set at p < 0.05.

Results

Included and Excluded Studies

A total of 34 full-text articles were assessed to figure out the eligible studies. Among them, 29 studies (27 case reports and two case series) that recruited 32 patients were considered eligible for inclusion [12-40], while the remaining five were excluded with reasons [41-45]. The study by Kishino et al. was excluded due to the fact that the hemorrhagic peritoneal fluid was attributed to retrograde menstruation, whereas the study by Bean et al. was excluded due to insufficient data [41,45]. More specifically, three studies were excluded due to the fact that the full text could not be reached despite multiple attempts to contact the journal and authors [42-44].

Patient Characteristics

The median age of the 32 included patients was 32 years (range: 21-46 years). Data concerning ethnicity was available for 14 patients. More specifically, nine patients were of African origin (African-American, Afro-Brazilian, Afro- Caribbean, and Nigerian), while two patients were Caucasian, one was Hispanic, and two were Asian. Regarding the 26 patients with parity information available, 19 patients were nulliparous, whereas four were primiparous, and the remaining three were multiparous. Six patients reported a previous history of endometriosis, five of whom underwent an exploratory laparoscopy for the diagnosis and management of the disease. The median CA 125 values were 184 U/L (range: 22 to >5,009), as reported by 16 studies, while the median values for hemoglobin were 9.8 g/dL (range: 6.9-12.9 g/dL), which were data from 12 studies. Six patients were diagnosed with the presence of concomitant pleural effusion. Abdominal distention and progressively worsened discomfort were reported as the main symptoms, followed by abdominal pain, weight loss, anorexia, fever, nausea, and breathing difficulty (Table 1).

**Table 1:** Main characteristics of the included studies R: right, RUQ: right upper quadrant, EM: endometriosis, G: gravidity, P: parity, Hb: hemoglobin, N/A: not available, COC: combined oral contraceptives

Author and year	Age	Ethnicity	History of EM	G/P	Pleural effusion	CA 125 (U/mL)	Hb (g/dL)	Clinical symptoms	Clinical examination findings	Diagnosis (imaging or drainage)
											Bhojawala et al. (2000) [12]	34	Black	No	G0P0	Yes	N/A	11.4	Abdominal distension (four months), malaise, loose stools, nausea and vomiting (two weeks), shortness of breath, appetite loss	Tense and distended abdomen, hyperactive bowel sounds, positive fluid thrill	Laparotomy
Dias et al. (2000) [13]	41	Black	No	G0P0	No	N/A	N/A	No	N/A	Exploratory laparotomy
Cheong et al. (2003) [14]	41	Malay	No	P1	Yes	Normal	Normal	Worsening abdominal distension	Gross ascites	Paracentesis
Goumenou et al. (2006) [15]	46	N/A	Yes, laparoscopy (30 years old), infertility	G3P0	Yes, bilateral	3,504	10.2	Progressive dyspnea, abdominal distension, nausea, 7 kg weight loss	Tachypnea, ↓breath sounds, abdominal distension, fever	Thoracocentesis, paracentesis
Alabi et al. (2007) [16]	30	Black African	Yes, vaginal EM, six months, GnRH analog and goserelin	N/A	No	56	8.5	Abdominal distension and pain during IVF treatment with GnRH agonist	N/A	Paracentesis
Palayekar et al. (2007) [17]	N/A	African- American	No	P1	No	33.6	N/A	Abdominal distension, anemia	Moderate abdominal distension	Paracentesis
Santos et al. (2007) [18]	40	Brazilian	Yes, laparoscopy (longstanding amenorrhea)	G0P0	No	N/A	N/A	Upper abdominal pain, vomiting and weight loss of 11 kg, anemia	N/A	Paracentesis
Sait (2008) [19]	26	N/A	No	P0	No	3,140	N/A	Increased abdominal girth	Distended abdomen	Laparotomy
Ussia et al. (2008) [20]	26	Caucasian	Yes, thoracic and diaphragmatic	G0P0	No	Ν/A	N/A	Ascites	N/A	Laparoscopy
	23	N/A	No	G0P0	Yes	N/A	N/A	Severe dysmenorrhea and menstrual R shoulder pain	N/A	Thoracocentesis (twice)
Day et al. (2009) [21]	24	N/A	No, EM-related symptoms	G0P0	No	N/A	10.7	Two-year abdominal pain, nausea, vomiting, constipation, infertility	N/A	Paracentesis turbid brown fluid
Lin et al. (2010) [22]	29	N/A	No	G2P2	Yes	N/A	12.9	Light-headedness, palpitations	Hypovolemic shock	Paracentesis
Suchetha et al.(2010) [23]	36	N/A	No	Parous	No	>5,000	N/A	Massive ascites	Nodularity in Douglas	Paracentesis, laparotomy
Fernandes et al. (2011) [25]	28	Afro-Brazilian	No	G0P0	No	N/A	9.5	Progressive increase in abdominal girth, weight loss	Distended, nontender abdomen, positive shifting dullness	Paracentesis
Shabeerali et al. (2012) [24]	28	N/A	No	N/A	No	N/A	N/A	Abdominal distension (five weeks)	Ascites and mild tenderness	Paracentesis
	30	N/A	No	P2	No	96	N/A	Progressive abdominal distension and weight loss	N/A	Paracentesis
	40	N/A	No	G6P4	No	N/A	N/A	Ascites	Ascites	Paracentesis
Morgan et al. (2013) [26]	27	African	Yes, COC	G0P0	No	N/A	7	R neck and flank pain, light-headedness, and palpitations	Mildly distended abdomen, tender in the RUQ	Paracentesis
Mumtahana et al. (2014) [27]	36	Chinese	Yes	G0P0	No	78.23, 86.6, 5,009	N/A	Ascites, anemia	Abdominal distension	Paracentesis
Appleby et al. (2014) [28]	34	Nigerian	No	N/A	No	N/A	9.6	Abdominal distention, 4 kg weight loss	Gross ascites	Drainage
Asano et al. (2014) [29]	35	Japanese	No	G0P0	No	22	10	Dysmenorrhea, abdominal distention	Abdominal distention	Drainage
Bignall et al. (2014) [30]	36	Afro- Caribbean	No	G0P0	No	1123	10.8	Seven-month dysmenorrhea, deep dyspareunia, constipation	Abdominal tenderness and distention	Paracentesis
Cosma et al. (2014) [31]	36	N/A	Deep pelvic EM	N/A	No	184	N/A	Dysmenorrhea, dyschezia, epigastric menstrual pain	N/A	Drainage
Hasdemir et al. (2014) [32]	32	N/A	Yes, EM (laparoscopic biopsies)	N/A	Yes	47	N/A	Abdominal distension and shortness of breath	Massive ascites	Laparoscopy, drainage
Hinduja et al. (2015) [33]	34	N/A	No	P1A1	No	N/A	N/A	Abdominal bloating	N/A	Transvaginal aspiration of Douglas
Setubal et al. (2015) [40]	26	Caucasian	No	G0P0	No	100	N/A	Upper abdominal pain and distention	N/A	Paracentesis
Dun et al. (2016) [34]	26	Nigerian	Yes	P0	No	N/A	N/A	Ascites	N/A	Drainage
Pereira et al. (2017) [35]	21	N/A	No	G0P0	No	N/A	7.5	Abdominal distension, dyspnea	N/A	Laparoscopy
Magalhães et al. (2018) [36]	28	N/A	No	N/A	No	107.8, 889.6	N/A	Wasting syndrome, ↑abdominal girth, shortness of breath,c↓appetite	N/A	Diagnostic laparoscopy
Pang et al. (2019) [37]	40	N/A	No	G1P0	No	372.4	N/A	Lower abdominal pain, pelvic mass, dysmenorrhea	Palpable pelvic mass	Laparoscopy
Wang et al. (2019) [38]	24	Nigerian	No	G0P0	No	41.54, 113	6.9	Rapidly enlarging abdominal distension	Massive ascites	N/A
Gonzalez et al. (2020) [39]	32	Hispanic	Yes, massive hemorrhagic ascites	Null	N/A	N/A	N/A	Malaise, abdominal distension, loss of appetite, diffuse abdominal pain, breathing difficulty	N/A	Paracentesis

Additionally, endometriosis-related symptoms including dysmenorrhea, dyspareunia, and dyschezia were also recorded. Clinical examination revealed abdominal tenderness and distention with shifting dullness in palpation, palpable pelvic mass if present, and diminished breath sound in patients with simultaneous pleural effusion. In critically ill patients, signs of hemodynamic instability were also noted. In 19 cases, the diagnosis was established with an examination of the percutaneously drained HA, while in one patient, a transvaginal paracentesis through the pouch of Douglas was performed. Five patients underwent an exploratory laparoscopy and drainage, whereas an open surgical approach was applied to three women.

Quality Assessment

Based on the type of the included clinical cases, we considered the score of 5 points as the highest that could be assessed when excluding the three questions (from 4 to 6) from the quality assessment tool that attributed to cases of adverse drug events. A mean score of 3.5 (SD: ±0.85) was calculated, whereas the overall judgment on the quality of the recruited studies was that they were of moderate quality.

Main Outcomes

The median amount of fluid drained was 4,200 mL (range: 1,500-9,400 mL), and four patients underwent two or more sessions of paracentesis. Concomitant thoracentesis was performed three patients due to pleural effusion. The main treatment modalities included hormonal therapy, other medications for symptomatic relief, and surgical procedures. Various hormonal modalities were adopted, including GnRH agonists/analogs (goserelin and leuprorelin), combined oral contraceptives (COC), luteinizing hormone (LH) agonists, dienogest, medroxyprogesterone, and norethindrone. GnRH agonist treatment was used in 17 patients, GnRH antagonists in one patient, COC in three patients, LH agonist in one patient, dienogest in two patients, and medroxyprogesterone and norethindrone in one patient. There is a case that was treated with chemotherapeutic agents for suspected ovarian cancer [15] and two cases that were initially treated with antituberculous agents for suspected tuberculous ascites [24,38]. Therapy with fertility-preserving management was decided in all but five patients at the initial management and included resection of all visible endometriotic nodules, adhesiolysis, and respective repairs of the affected organs such as colectomies and anastomosis, as shown in Table 2. However, fertility was finally preserved in 27 patients. Seven patients underwent bilateral salpingo-oophorectomy with hysterectomy along with excision of all macroscopic pelvic endometriotic nodules and other procedures including omentectomy, appendectomy, and lymphadenectomy (Table 2). In 13 patients, an open approach was applied, whereas 24 patients had laparoscopic procedures. Six of them underwent both laparoscopic and laparotomic evaluation. Pregnancy outcomes were available for two patients who achieved a single and twin pregnancy [30,40]. Both of them conceived with the use of in vitro fertilization (IVF) techniques and delivered preterm through cesarean section at 32 and 35 weeks of gestation, respectively. Two of the patients had postoperative ileus; among them, one died due to peritonitis and sepsis after intestinal obstruction and enterocutaneous fistulae.

**Table 2:** Main outcomes N/A: not available, EM: endometriosis, R: right, L: left, PO: postoperative, wk: week, mo: months, yr: year, TAH: total abdominal hysterectomy, BSO: bilateral salpingo-oophorectomy, USO: unilateral salpingo-oophorectomy, COC: combined oral contraceptive, CS: cesarean section, NED: no evidence of disease, DOD: die of disease, DIE: deep infiltrating endometriosis, DNG: dienogest

Author and year	Amount of fluid drained	Management		Histology	Follow-up (recurrence-reoperation)
		Primary treatment	Secondary treatment		
Bhojawala et al. (2000) [12]	9,000	Laparotomy, TAH-RSO, adhesions	N/A	Endometriosis of the cervix, R fallopian tube, and ovary	One mo - R exploratory thoracotomy, decortication of the R lung, and parietal pleurectomy; six wks - NED
Dias et al. (2000) [13]	N/A	GnRH analog	N/A	N/A	Six mo - progressive ↓ of ascites
Cheong et al. (2003) [14]	5,600	Exploratory laparotomy-peritoneal biopsies	Yes, medical	EM	N/A
Goumenou et al. (2006) [15]	4,000	First-line chemotherapy (carboplatin/taxol), suspected malignancy	Two mo - exploratory laparotomy debulking/TAH-BSO, omentectomy, appendectomy, biopsies, L pelvic lymphadenectomy	Inflammation and EM	Six mo - NED
Alabi et al. (2007) [16]	5,000	Emergent diagnostic laparoscopy, extensive pelvic EM including the bowel	Second laparoscopy after one wk, adhesiolysis, and bowel mobilization	EM	Two mo - ascites (2.5 L), recurrence; one mo - laparoscopy multiple biopsies; spontaneous conceive
Palayekar et al. (2007) [17]	4,000-6,000	Exploratory laparotomy - advanced pelvic EM, TAH-BSO	Declined hormonal therapy	EM	12 mo - NED
Santos et al. (2007) [18]	N/A	Laparoscopy (nondiagnostic), laparotomy - adhesiolysis, encapsulating peritonitis	N/A	EM	Five mo - intestinal obstruction, enterocutaneous fistulae, DOD (peritonitis and sepsis)
Sait (2008) [19]	5,000	Laparotomy - bilateral ovarian cystectomy, multiple biopsies	GnRH analog for six mo, maintenance with COC	EM	12 mo - NED
Ussia et al. (2008) [20]	1,000, >1,000, 2,000, 1,500	Three laparoscopies during three yrs, two mo laparotomy - massive adhesiolysis, appendicectomy, omentectomy, USO	GnRH	EM	36 mo - NED
	1,500	Laparoscopy - ascites, frozen pelvis, bowel adhesions, and EM spots; second laparoscopy (one yr after GnRH agonist) - ascites, adhesions, DIE, rectovaginal nodule excision, ureterolysis, resection sigmoid anastomosis	GnRH agonist and intermittent corticosteroids	EM	NED
Day et al. (2009) [21]	4,000	Exploratory laparoscopy - stage IV ASRM EM, multiple biopsies	Leuprolide acetate 11.25 mg	EM	Ileus PO (44-d admission - conservative management), three mo - NED
Lin et al. (2010) [22]	2,000	Diagnostic laparoscopy - electrocauterization EM of the L broad ligament	N/A	N/A	N/A
Suchetha et al.(2010) [23]	6,000	Diagnostic laparotomy - abdominal cocoon, biopsies of the adnexa, bladder, peritoneum, omentum, and stomach	One yr - leuprolide		Three mo - bilateral ovarian masses, hydronephrosis -omentectomy
Fernandes et al. (2011) [25]	9,400	Laparoscopy - adhesions, mesosigmoid biopsy	Three mo - GnRH analog estrogen and then continuous estrogen-progestin	Fibrosis and extensive hemosiderin deposition, endometrial glands and stroma	12 mo - NED
Shabeerali et al. (2012) [24]	N/A	Diagnostic laparoscopy conversion to laparotomy, dense adhesions with small and large bowel, biopsies; second operation TAH-BSO	One yr - GnRH analogs (partial response), TAH-BSO	N/A	12 mo - NED
	N/A	Laparoscopy - ascites, peritoneal biopsies	Subtotal hysterectomy and BSO	EM	12 mo - NED
	2,500, 3,000	Two laparoscopies - suspected tuberculosis (antituberculosis treatment); third laparoscopy - ascites, adhesions, biopsies	GnRH analogs	Endometrial glands and endometrioid stroma	NED
Morgan et al. (2013) [26]	4,500	Leuprolide	N/A	N/A	N/A
Mumtahana et al. (2014) [27]	3,000, 2,500	Exploratory laparoscopy, dense adhesions, bilateral ovarian masses, Douglas nodules	Goserelin acetate/mo	EM	NED
Appleby et al. (2014) [28]	N/A	Laparoscopy - endometrial ovarian and fallopian tube deposits (biopsies)	GnRH antagonist	EM	Six mo - NED
Asano et al. (2014) [29]	5,500	Exploratory laparotomy - adhesions, biopsies of brown omental nodules stage IV EM	Eight y - GnRH agonist and ascites drainage (13 times) - switch to DNG	EM	12 mo - NED
Bignall et al. (2014) [30]	3,500, 1,600	Laparoscopy - biopsies of uterosacral ligament and bowel nodules stage IV EM	GnRH analogs	Cyclical endometrium in proliferative phase	Pregnancy achieved (IVF) - live birth at 32 wks emergent CS/two wks recurrent ascites - 5 GnRH injections NED
Cosma et al. (2014) [31]	4,200, 250	Laparoscopy - adhesions, excision of pelvic EM, colectomies, three anastomoses, and temporarily ileostomy	Second-look laparoscopy and ileostomy closing (22 days)	EM	48 mo - NED
Hasdemir et al. (2014) [32]	2,500	Paracentesis and six mo leuprorelin	N/A	EM by paracentesis	Three mo - recurrence - DNG
Hinduja et al. (2015) [33]	4,500, 2,500, 3,000, 4,000, 3,500	Diagnostic laparoscopy - biopsies of omental and bowel nodules	Three mo - leuprolide 3.75 mg	EM	Six mo - multiple recurrences of ascites, recurrence of ascites after TAH-BSO with vaginal discharge/one y - NED
Setubal et al. (2015) [40]	2,500, 1,000	Diagnostic laparoscopy - pelvic adhesions, rectal and ovarian implants, omental retractions, hematic liver implants, multiple biopsies	Three mo - COC	EM	Three mo - ascites recurrence-GnRH agonist; second laparoscopy - DIE, GnRH agonist; pregnancy achieved, live birth of twins at 35 weeks/NED on COC
Dun et al. (2016) [34]	7,000, 7,800	Exploratory laparotomy - biopsies	Three mo - goserelin and oral and one y oral medroxyprogesterone	EM	Three mo - recurrence, unsuccessful conceive attempt; laparoscopy, EM resection with peritoneal stripping, laser excision, ablation; six mo - NED
Pereira et al. (2017) [35]	4,000	Laparoscopy (third laparoscopy) - extensive EM adhesions in the pelvis, bipolar and monopolar excision of EM	Monophasic oral contraceptive pills	EM	NED
Magalhães et al. (2018) [36]	8,000	Diagnostic laparoscopy - multiple adhesions and encapsulating peritonitis (nondiagnostic); second laparoscopy - biopsies	Goserelin acetate	Chronic peritonitis and hemosiderin deposits	Six mo - NED
Pang et al. (2019) [37]	2,000	Laparoscopy converted to laparotomy (bleeding) - TAH BSO, R broad ligament mass excision	No	Mass with a monolayer of normal-looking endometrial glands and stroma	Three mo - NED
Wang et al. (2019) [38]	N/A	GnRH analogs (leuprorelin) for three mo and then droperidoland ethinyl estradiol tb for eight mo	No	Endometrial glandular cells and surrounding stromal cells (core needle biopsy of the omentum)	Five mo - stable ascites -symptom improvement

Discussion

In the present study, we analyzed the characteristics of 32 women with EM-related hemorrhagic ascites. The majority of patients were nulliparous, while abdominal distention and progressively worsened discomfort were recorded as the main symptoms at presentation. The mean amount of drained ascitic fluid was 4,200 mL. The treatment options included not only medical-hormonal but also surgical therapeutic modalities. Fertility preservation was achieved in 27 patients, while two of them achieved pregnancy with IVF techniques. Two cases of postoperative ileus were reported and one postoperative death due to peritonitis.

The role of elevated CA 125 levels is debatable; there have been reports indicating elevated CA 125 levels in patients with ascites that are non-cancer-related, such as cirrhotic or even in heart failure [46,47]. According to the findings of the present study, CA 125 levels ranged from 22 to 5,000, which could be considered conflicting given the high suspicion of malignancy in patients with ascites and elevated CA 125 levels. Furthermore, before confirming the presence of ascites with ultrasound, there are also some percussion signs including puddle signs, floating ice, and flank dullness that could be useful [48]. The reported overall accuracy of physical examination maneuvers is approximately 58%, with sensitivity and specificity ranging from 50% to 94% and from 29% to 82%, respectively [49].

The differential diagnosis of a woman who presents with ascites is relatively challenging. Besides hepatic and renal failure, which are considered the main causes of the formation of ascites, malignant and infectious intra-abdominal diseases are also responsible for the concentration of diffusion of peritoneal fluid rich in proteins [50]. With regard to malignant diseases, epithelial ovarian and tubal cancer, primary peritoneal serous carcinoma, and endometrial cancer can be associated with ascites formation [51]. Furthermore, benign ovarian cysts, endometriosis, ovarian hyperstimulation syndrome, peritoneal tuberculosis, and Meigs syndrome should also be considered in the differential diagnosis of female ascites [51].

Endometriosis-related ascites can be easily misdiagnosed as ovarian cancer-related due to the fact that both entities share some similar symptoms. To that end, hemorrhagic endometriotic ascites can present with abdominal distention and pain, loss of appetite, and weight loss, mimicking atypical cancer symptoms. However, careful evaluation of patients’ medical history and endometriosis-related symptoms such as dysmenorrhea, dyspareunia, and cyclical pain should be thoroughly investigated. Furthermore, high clinical suspicion should be paid to the cases of malignancy arising from endometriosis [52]. The prevalence of malignancy is about 0.7%-1.6% in patients with endometriosis [52]. Consequently, the exclusion of malignancy is of critical importance, and thus, it is considered safer to set the final diagnosis after surgical evaluation and histological examination of the excised specimens. In that setting, some of the patients included in the present study underwent a diagnostic laparoscopy with a concomitant aspiration of the ascitic fluid and peritoneal biopsies. The percutaneous aspiration of the ascites has also been applied in some cases. This first-line diagnostic modality is an easy-to-perform bedside practice and can facilitate a more accurate further management of the disease [53]. The cytological findings of the aspired ascitic fluid can reveal epithelial and stromal cells in a hemorrhagic environment with hemosiderin and hemofuscin-laden macrophages [53,54].

There are some reports available in the literature indicating the concomitant detection of encapsulating peritonitis in patients with endometriosis-related ascites. Encapsulating peritonitis, also known as abdominal cocoon or frozen ascites, is a rare entity defined as the formation of a thick fibrin membrane that entraps the bowel loops [36]. According to a recent systematic review by Magalhães et al. on endometriosis-related ascites and encapsulating peritonitis, only six cases of endometriosis-associated encapsulating peritonitis have been recorded in the literature [18,36]. Additionally, another case of encapsulating peritonitis has been recently published by Gonzalez et al. and was attributed to recurrent HA due to endometriosis [39]. A potential theory supports that endometriosis-related inflammation causes peritoneal irritation and further enhances fibrosis and inflammation, resulting in the formation of encapsulating peritonitis.

The exact pathophysiology of the formation of endometriosis-related ascites still remains ill-defined. Bernstein et al. were the first to study on the pathogenesis of endometriosis-associated ascites. The authors claimed that the presence of endometrial cells in the peritoneal cavity under unknown mechanisms can activate the peritoneal cells to produce ascitic fluid [54]. Additionally, another theory suggested the peritoneal irritation from the spontaneous rupture of endometriotic cysts, which can produce reactive peritoneal fluid [54]. Another potential mechanism is based on the inflammatory response caused by the effect of the uterine hormones on the ectopic endometriotic lesions [55]. The aforementioned theories are well supported by recent studies speculating on the diversity of the biochemical and metabolic profiles of the peritoneal fluid in patients with endometriosis. More specifically, according to Polak et al., the hemoglobin levels in the peritoneal fluid of patients with endometriosis were significantly elevated compared to both controls and women with ovarian cysts, while, interestingly, antioxidant parameters were significantly lower in patients with endometriosis, creating an oxidative intraperitoneal environment [56-58].

The outcomes of the present study indicated a high prevalence of HA in patients of African origin. A respective high prevalence was also observed in the systematic review by Gungor et al. who reported a proportion of more than 60% of African ethnicity among women with endometriosis-related ascites [54]. Little is known with regard to the potential association between endometriosis and race. Despite the fact that the currently available literature provides evidence of a higher prevalence of endometriosis in White women compared to African, those reports are subjected to significant bias related to diversity in socioeconomic status, access to the healthcare system, and childbearing age [59]. Additionally, Bougie et al. highlighted the potential diversity of symptoms and clinical presentation of endometriosis among different ethnicities, which could also explain the elevated prevalence of HA among African populations with endometriosis [59,60].

Concerning the management of endometriosis-associated ascites, it is mainly based on the extent of the underlying endometriosis and is that of endometriosis including surgery or medication or both. Additionally, the drainage of the ascetic fluid is crucial for the alleviation of abdominal distention and discomfort. Due to the fact that a significant proportion of patients (six in the present study) presented with concomitant pleural effusion, thoracentesis is also indicated for the symptomatic relief of breath discomfort. The majority of the patients in the present study underwent surgery for the management of endometriosis. The extent of surgical procedures is based on the age of the patient and the desire for fertility preservation [61]. Moreover, adjuvant pharmaceutical therapy was administered to 16 patients postoperatively. A favorable effect of postoperative medication maintenance therapy has been reported for symptomatic relief and recurrence prevention, but its exact role still remains controversial [61,62].

Limitations

Despite the plethora of reports, the true prevalence of HA could not be precisely reached since no observational studies are available in the field and thus precluded further research. The fact that our results are based only on case reports and two case series constitutes the main limitation of the study and precludes generalization of the conclusions and further quantitative and qualitative analysis. In addition to this, there is no sufficient evidence concerning the pathophysiology of ascites formation, while it is not clear for all cases whether the bloody peritoneal fluid was concentrated after the rupture of an ovarian endometrioma or whether other mechanisms similar to those forming malignant ascites are involved. Finally, there is significant heterogeneity in the included studies, and some parameters were omitted by some studies, which was another limitation and precluded reaching firm results.

## Conclusions

The present review accumulates the current knowledge with regard to the natural history, characteristics, and management of adult females who presented with hemorrhagic ascites due to endometriosis. The differential diagnosis of a woman who presents with ascites is relatively challenging. Endometriosis-related hemorrhagic ascites is a relatively rare expression of the disease. Nonetheless, it should be considered in the differential diagnosis of women with ascites and clinical suspicion of endometriosis. Additionally, the exclusion of malignancy is considered of critical importance. High clinical suspicion should be paid to cases of malignancy arising from endometriosis. The exact pathophysiologic pathways of endometriotic hemorrhagic ascites formation still remain elusive, despite the plethora of available theories.

The management of hemorrhagic ascites should speculate on both alleviation of the abdominal distention due to the presence of ascites and treatment of the underlying disease. The currently available literature is limited to case reports and case series, thus precluding reaching firm conclusions. Further research in the field is needed to decode the pathophysiology of the disease and decide on the optimal treatment.
